# Supplementary material for: Co-Designed Cardiac Rehabilitation for the Secondary Prevention of Stroke (CARESS): A Pilot Program Evaluation
Source: Healthcare (Basel). 2024 Apr 3;12(7):776. doi: 10.3390/healthcare12070776 (PMC11012137; doi:10.3390/healthcare12070776)
Supplement: Supplementary file 1 [file healthcare-12-00776-s001.zip › healthcare-2902488-supplementary.pdf]

Online supplement

Supplementary Table S1. Questions used for co-design workshops

People with lived experience of stroke	Health professionals
What are your views of care for stroke patients after discharge, particularly in relation to risk factor management?	What are your views of care for stroke patients after discharge, particularly in relation to risk factor management?
What are your views on our proposed solution of adapting the current cardiac rehabilitation program to use in stroke survivors?	What factors contribute to this being an issue?
How well would this program suit your needs?	What are your views on our proposed solution of adapting the current cardiac rehabilitation program to use in stroke survivors?
What are the strengths of the proposed program?	Are there particular things we should understand to make the program suitable for people with stroke?
What would assist you in being able to attend this type of program?	What are the strengths in the existing system that could help us to implement this solution?
What would you change to make it better for people that have had a stroke?	

Brief Participant Information Sheet for recruitment

Pilot study of Cardiac Rehabilitation for the Secondary Prevention of Stroke (CARESS)

What is the study about?

We are testing a new program for people that have had a stroke. The program is based on cardiac rehabilitation programs that people attend after they have had a heart attack. This study has been approved by the Tasmanian Health and Medical Human Research Ethics Committee (H0017731)

The aim of the study is to examine whether a comprehensive lifestyle and risk factor management program developed for people after they have had a stroke is feasible, acceptable and associated with positive changes in their risk factors for stroke, quality of life and fitness.

What does the study involve?

The program will be run by a registered nurse with support from specialists in exercise and rehabilitation. It will include of exercises for 1 hour in a gym and group discussion for 1 hour about topics relevant to people that have recently had a stroke. Support people (e.g. spouse or child) will be encouraged to attend the group discussion. It will run for 7 weeks at the Menzies Institute for Medical Research in their clinical research facility.

Before the program we will take measurements of risk factors for stroke, fitness, quality of life and psychological wellbeing. This will take around 1 hour. We will repeat these assessments 1 week and 6 weeks after the program. We will use the test results to see if the program has changed any of these factors. We will also ask you about your experience with the program, including what you thought was good and bad. Support people will be asked to complete a brief assessment (around 15 minutes) about their quality of life and knowledge of stroke before and after the program.

We will use this information to develop a larger study to test whether the program is effective. The results will be used in research papers and presentations to tell people what we have found.

What are the risks of being in the study?

The program includes exercise, and this can be associated with risks including falling, muscle soreness or injury. Large studies of people with stroke have found that exercise is safe with only small numbers of people suffering these events. The study nurse, doctors and other health professionals will be monitoring participants carefully to reduce the risk of these events.

The program includes discussion in a group about you and your life after your stroke. This might cause you some distress or make you feel uncomfortable. If this happens you are able to stop participating in the discussion and we can arrange for support from a qualified person if needed.

What are the benefits of being in the study?

As this is a new program we cannot guarantee that you will benefit. You might get a better understanding of stroke. You may experience positive effects on your health and wellbeing from the exercise program. This is a short term study so the effects might be small. You will meet other people that have had a stroke and this type of support can have benefits for wellbeing.

What should I do if I would like to participate?

You should ask for the full information sheet and consent form. Please read it carefully and ask any questions you have. A study team member will check your eligibility and if you are eligible you can

be scheduled for the initial assessments of your health. If you have questions, you can call the study Chief Investigator Dr Seana Gall on 6226 4728 or email Seana.Gall@utas.edu.au

Supplementary Table S2. Assessments undertake before, 1 week and 6 weeks after the program

**Outcome**	**Pre-program**	**1 week after completion**	**6 weeks after completion**
Demographics*	Yes	No	No
Education level*	Yes	No	No
Smoker	Yes	Yes	Yes
Height	Yes	No	No
Weight	Yes	Yes	Yes
Diet[1] and physical activity[2]	Yes	Yes	Yes
Assessment of Quality of Life (AQoL)*[3]	Yes	Yes	Yes
Fatigue Assessment Scale[4]	Yes	Yes	Yes
Depressive symptoms - Patient Health Questionnaire[5]	Yes	Yes	Yes
Cholesterol with point of care measure	Yes	Yes	Yes
Grip strength with dynamometer	Yes	Yes	Yes
Functional capacity with 6 min walk test	Yes	Yes	Yes
FICSIT-4 Balance Scale	Yes	Yes	Yes
Blood pressure	Yes	Yes	Yes
Participant satisfaction[6]	No	Yes	Yes
Stroke knowledge -stroke awareness scale*[7]	Yes	Yes	Yes

* also assessed in carers/spouses/partners of participants

Exercise session planning and delivery

Prior to each exercise session, an exercise physiologist, a physiotherapist and, if necessary, a neurologist, would plan the program for that week, accounting for the progression, regression, or modification of the circuit of exercises to suit each participant. Each exercise session was comprised of a 15 minute warm up, followed by a 30 minute low to moderate intensity exercise circuit, concluding with a 15 minute cool down. The circuit included aerobic and resistance exercises, all of which could be completed as standing or sitting versions. A list of the exercises is provided in a table in Appendix 1. Ongoing monitoring took place throughout the exercise sessions, using continuous heart rate monitoring, and self-reported perceived exertion using the Borg Scale, for each exercise. Exercises were progressed according to the judgement of the clinical staff, partly on the basis of this monitoring. Information was elicited about any adverse events that had taken place between sessions. Any adverse events that occurred during exercise sessions were recorded using a standard adverse event reporting form.

Supplementary Table S3. Recommended Aerobic and Resistance Training Exercises in CARESS program

Recommended exercise	Exercise and progression
**Aerobic exercises**	
Exercise bike	Time, resistance, speed
Treadmill	Time, angle, speed
Cross-trainer	Time, resistance, speed
Step ups	Speed
Sit to stand	Speed
**Resistance exercises**	
Lower limb exercises	
Sit to stand	Weighted vest, hand weights, theraband, hands, no hands
Squats	Wall, fit ball, vest, hand weights, single leg
Step ups	Weighted vest, hand weights, height
Lunges	Weighted vest, hand weights, back wards, lateral, diagonal
Leg press	Increase machine weight
Hip abduction/extension	Free or machine weights
Calf raises	Weighted vest, hand weights, single leg, machine
Upper limb exercises	
Push ups	Wall, side lying, knees on or off ground
Chest press	Free weights or machine weights
Shoulder press	Free weights or machine weights
Bicep curls	Free weights
Triceps	Theraband, free weights or machine
Lats	Seated row, lat pull down

Supplementary Table S4. Group discussion topics informed by co-design workshops

Week	Topic	Resources
1	Life after stroke – risk factors, stroke signs, recurrent stroke, online tools	My Stroke Journey<br>EnableMe
2	Risk Factors – major modifiable risk factors, risk factor checklist, healthy lifestyle goalsetting	My Stroke Journey<br>EnableMe<br>Quit Tasmania<br>Eat Well Tasmania
3	Mental Health – depression, anxiety, fatigue, personality change after stroke, and management strategies	Stroke Foundation handouts on depression and anxiety, personality and emotional changes, and fatigue after stroke
4	Medications – common stroke medications, medication management strategies, pharmacological management of pain	Handouts on medications after stroke
5	Getting Back to Everyday Activities – return to work, driving, and activities of daily living, including strategies for adaptation and assistance	Handouts on return to work, and return to driving
6	Relationships After Stroke – possible effects of stroke on relationships and management strategies	Handouts on sex and relationships after stroke, and supporting children and family members after stroke
7	Memory and Thinking – possible effects of stroke on memory and cognition, and management strategies and relevant services	Handouts on thinking and perception after stroke, communication after stroke, and vision after stroke

Supplementary Table S5. Stroke knowledge before and after CARESS program completion

Questions		Baseline (n=10)	Week 1 (n=8)	Week 6 (n=8)
Can you tell me what you understand by a "stroke"?	Blood clot in the brain	7 (70%)	6(75%)	1(12.5%)
	Brain haemorrhage	0	0	2(25%)
	A condition that affects the brain	3(30%)	2(25%)	4 (50%)
	Circulation problem in the brain	2(20%)	1 (12.5%)	
	Don't know	0	0	
	Other	0	0	1(12.5%)
What do you believe are the risk factors associated with stroke?	Stress	1(10%)	3 (37.5%)	3 (37.5%)
	High blood pressure	2(20%)	3 (37.5%)	7 (87.5%)
	High cholesterol	1(10%)	2 (25%)	4 (50%)
	Smoking	5 (50%)	3 (37.5%)	5 (62.5%)
	Diabetes	0	1 (12.5%)	0
	Overweight	4(40%)	4 (50%)	5 (62.5%)
	Drinking alcohol	2(20%)	2 (25%)	2 (25%)
	Lack of exercise	4(40%)	7 (87.5%)	5 (62.5%)
	Increasing age	0	1 (12.5%)	0
	Hereditary family history	1(10%)	0	1 (12.5%)
	Other answers	6(60%)	2 (25%)	4 (50%)
	Don't know	0	0	0
What do you think are the symptoms or warning signs of a stroke?	Dizziness	2(20%)	1 (12.5%)	3 (37.5%)
	Difficulty understanding/sudden confusion	1(10%)	2 (50%)	3 (37.5%)
	Severe headache	1(10%)	1 (12.5%)	0
	Problems with vision	0	2 (25%)	3 (37.5%)
	Shortness of breath	0	0	0
	Slurred speech	1(10%)	4 (50%)	3 (37.5%)
	Weakness on one side of the body	2(20%)	5 (62.5%)	5 (62.5%)
	Facial Weakness/Fallen Face	3(30%)	3 (37.5%)	1 (12.5%)
	Any mention Face, Arm, Speech, Time (FAST)	2(20%)	2 (25%)	2 (25%)
	Numbness on one side of the body	1(10%)	0	0
	Other	3(30%)	1 (12.5%)	2 (25%)
	Don't know	0	0	1 (12.5%)

Questions		Baseline (n=10)	Week 1 (n=8)	Week 6 (n=8)
What is the FIRST thing that you would do if you thought that you were having a stroke?	Wait and see, for example lie down, try to relax, ignore it	1(10%)	1 (12.5%)	1 (12.5%)
	Tell someone, for example a family member, friend, or neighbor	2(20%)	2 (25%)	1 (12.5%)
	Take something, for example aspirin or a headache medication			
	Contact the GP			
	Drive or have someone drive me to the hospital			
	Call an ambulance	6(60%)	5 (62.5%)	6 (75%)
	Don’t know			
	Other answers	1(10%)		

Supplementary Table S6. Characteristics of historical control group

	**Baseline**		**Follow up**	
	**n/mean**	**%/sd**	**n/mean**	**%/sd**
Age *(years)* mean(SD)	67.8	12.73	67.8	12.73
Sex				
Men	18	53		
Women	16	47		
Type of stroke				
Ischemic	31	91		
Haemorrhagic	3	9		
missing				
Time since stroke (weeks)	6.44	(4.30)		
Cognition mean (SD)	22.07	5.64	22.7	5.64
5 times sit to stand test *(sec)* mean (SD)	19.04	21.04	17.32	14.13
Anxiety mean (SD)	5.8	5.13	6.20	4.3
Depression (z score) mean (SD)	-0.22	0.68	0.16	0.91
Fatigue score mean (SD)	22.3	7.8	23	8.0
6-minute walk mean distance mean *(m)* (SD)	280.1	165.1 (40-610)	347.2	181.9 (60-698)

1. Leppala J, Lagstrom H, Kaljonen A, Laitinen K. Construction and evaluation of a self-contained index for assessment of diet quality. Scand J Public Health. 2010 Dec;38(8):794-802.
2. Craig CL, Marshall AL, Sjostrom M, Bauman AE, Booth ML, Ainsworth BE, et al. International physical activity questionnaire: 12-country reliability and validity. Medicine and science in sports and exercise. 2003 Aug;35(8):1381-95.
3. Richardson J, Iezzi A, Khan MA, Maxwell A. Validity and reliability of the Assessment of Quality of Life (AQoL)-8D multi-attribute utility instrument. The patient. 2014;7(1):85-96.
4. Cumming TB, Mead G. Classifying post-stroke fatigue: Optimal cut-off on the Fatigue Assessment Scale. J Psychosom Res. 2017 Dec;103:147-49.
5. Towfighi A, Ovbiagele B, El Husseini N, Hackett ML, Jorge RE, Kissela BM, et al. Poststroke depression: a scientific statement for healthcare professionals from the American Heart Association/American Stroke Association. Stroke. 2017;48(2):e30-e43.
6. Dyer N, Sorra JS, Smith SA, Cleary PD, Hays RD. Psychometric properties of the Consumer Assessment of Healthcare Providers and Systems (CAHPS®) Clinician and Group Adult Visit Survey. Med Care. 2012 Nov;50 Suppl(Suppl):S28-34.
7. Hickey A, Holly D, McGee H, Conroy R, Shelley E. Knowledge of Stroke Risk Factors and Warning Signs in Ireland: Development and Application of the Stroke Awareness Questionnaire (SAQ). International Journal of Stroke. 2011 2012/06/01;7(4):298-306.
